# Immune profile of primary and recurrent epithelial ovarian cancer cases indicates immune suppression, a major cause of progression and relapse of ovarian cancer

**DOI:** 10.1186/s13048-023-01192-4

**Published:** 2023-06-15

**Authors:** Pavan Kumar, Samruddhi Ranmale, Sanket Mehta, Hemant Tongaonkar, Vainav Patel, Amit Kumar Singh, Jayanti Mania-Pramanik

**Affiliations:** 1 ICMR-National Institute for Research in Reproductive and Child Health, Jehangir Merwanji Street, Parel, Mumbai, 400012 India; 2 Saifee Hospital, Mumbai, 400004 India; 3 P. D. Hinduja National Hospital & Medical Research Centre, Mumbai, 400016 India

**Keywords:** Epithelial ovarian cancer, Immune cell receptors, Cognate ligands, Cytokines, Tumor microenvironment

## Abstract

**Background:**

Ovarian cancer is the third most prevalent cancer in Indian women. Relative frequency of High grade serous epithelial ovarian cancer (HGSOC) and its associated deaths are highest in India which suggests the importance of understanding their immune profiles for better treatment modality. Hence, the present study investigated the NK cell receptor expression, their cognate ligands, serum cytokines, and soluble ligands in primary and recurrent HGSOC patients. We have used multicolor flow cytometry for immunophenotyping of tumor infiltrated and circulatory lymphocytes. Procartaplex, and ELISA were used to measure soluble ligands and cytokines of HGSOC patients.

**Results:**

Among the enrolled 51 EOC patients, 33 were primary high grade serous epithelial ovarian cancer (pEOC) and 18 were recurrent epithelial ovarian cancer (rEOC) patients. Blood samples from 46 age matched healthy controls (HC) were used for comparative analysis. Results revealed*,* frequency of circulatory CD56^Bright^ NK, CD56^Dim^ NK, NKT-like, and T cells was reduced with activating receptors while alterations in immune subsets with inhibitory receptors were observed in both groups. Study also highlights differential immune profile of primary and recurrent ovarian cancer patients. We have found increased soluble MICA which might have acted as “decoy” molecule and could be a reason of decrease in NKG2D positive subsets in both groups of patients. Furthermore, elevated level of serum cytokines IL-2, IL-5, IL-6, IL-10, and TNF-α in ovarian cancer patients, might be associated with ovarian cancer progression. Profiling of tumor infiltrated immune cells revealed the reduced level of DNAM-1 positive NK and T cells in both groups than their circulatory counterpart, which might have led to decrease in NK cell’s ability of synapse formation.

**Conclusions:**

The study brings out differential receptor expression profile on CD56^Bright^NK, CD56^Dim^NK, NKT-like, and T cells, cytokines levels and soluble ligands which may be exploited to develop alternate therapeutic approaches for HGSOC patients. Further, few differences in the circulatory immune profiles between pEOC and rEOC cases, indicates the immune signature of pEOC undergoes some changes in circulation that might facilitated the disease relapse. They also maintains some common immune signatures such as reduced expression of NKG2D, high level of MICA as well as IL-6, IL10 and TNF-α, which indicates irreversible immune suppression of ovarian cancer patients. It is also emphasized that a restoration of cytokines level, NKG2D and DNAM-1on tumor infiltrated immune cells may be targeted to develop specific therapeutic approaches for high-grade serous epithelial ovarian cancer.

**Supplementary Information:**

The online version contains supplementary material available at 10.1186/s13048-023-01192-4.

## Introduction

The burden of ovarian cancer is increasing globally. A 47% rise in ovarian cancer incidence is predicted by the year 2040. Second highest ovarian cancer incidences are reported in India whereas the mortality rate is highest in the world [1]. Relative frequency of high grade serous epithelial ovarian cancer in India is 87.6% which is also highest in the world [2]. Early diagnosis of the disease is rare due to its asymptomatic nature of growth and delayed onset of symptoms. The 5-year survival rate is approximately 25% for patients with high-grade serous epithelial ovarian cancer [3]. The major complication is the recurrence of the disease and acquired resistance towards standard platinum-based chemotherapeutic agents [4]. The outcome of the disease is heterogeneous even after considering the most common factors such as stage, grade, and response to therapy. This disparity is thought to be driven by both the tumor and the host [5]. Ovarian cancer is considered an immunogenic tumor. The tumor microenvironment is infiltrated by various immune and non-immune cells which is considered to be associated with the progression and metastasis of the disease [6, 7]. However, the role of natural killer (NK) cells in the prognosis of ovarian cancer is not yet clear. Based on the density of CD56 expression NK cells are dissected into CD3-CD56 + ^Bright^ and CD3-CD56 + ^Dim^ NK cell subsets. CD3-CD56 +^Bright^NK cells are efficient cytokine produces whereas CD3-CD56 + ^Dim^ NK cells are primarily responsible for cytotoxic activity [8]. Tumor associated NK cells with low level of activating receptors were associated with a poor overall survival of ovarian cancer patients. However, total CD56 + NK cells were associated with better progression free survival and overall survival of these patients [9]. Thus, specific phenotype of NK cells may determine the functional outcome. To combat tumor escape and relapse; NK cells can recognize the loss of HLA molecules for activation, induce inflammation and kill target tumor cells in an antigen-independent manner [10]. NK cells have various mechanisms to kill their target cells such as polarization and release of cytotoxic granules after activation signal; another mechanism is the use of death receptor-induced killing of target cells [11]. NK cells express a repertoire of germline-encoded activating and inhibitory surface receptors. Major families of these receptors are Killer immunoglobulin-like receptors (KIR), Natural cytotoxicity receptors (NCR), and CD94/NKG2 heterodimers that belong to the C-type lectin superfamily. Engagement of these receptors with their cognate ligand and fine balance of threshold of the inhibitory and activating signal determines the functional status of NK cells [12]. It is widely accepted that aberrant expression of these receptors may affect the disease outcome. Furthermore, T cells are the major adaptive immune cells perform response to pathogen, allergen and tumors [13]. T cells have been extensively studied in ovarian cancer [14]. Baseline infiltrations of T cells have played a key prognostic factor for different tumors. The presence of intratumoral CD8 + and CD4 + T cells is independently associated with a good prognosis of the disease. However, the antitumor effect of these immune cells is affected by their number, relative frequency, and functional capabilities [15]. In addition to CD3 + CD56-T cells, CD3 + CD56 + Natural killer T (NKT-like) cells are another important subset of highly differentiated, CD1d independent and MHC unrestricted T cells subset considered as a link between innate and adaptive immune systems [16]. However, the function of NKT- like cells is severely compromised in ovarian cancer patients, this impairment in NKT-like cells function is driven by soluble MHC class-1 related sequences (MICs) by down-regulating the prominent activating receptors NKG2D expressed over the surface of NKT-like cells [17]. Moreover, various lymphocyte subsets work co-operatively, which also decides the patient’s survival [18]. Although the normal expression of NK cell receptors is required for the optimum functioning of these cells, the function of these receptors is largely influenced by the level of cognate ligands as well. A large group of molecules (ligands) expressed over the surface of multiple tumor types are recognized by the activating NK cell receptors [19]. NCR receptors are important for the activation of NK cells and these receptors recognize ligands over malignant cells. NKp30 has two ligands B7H6 and BAG6 restricted over malignant cells, while the ligand for NKp46 is not known [20]. NKG2D is a dominant activating cell surface receptor recognizing the ligands MICA, MICB, and ULBPs, considered as a potential therapeutic target [21]. Ligands for activating receptor DNAM-1, Nectin-2/CD112, and PVR/CD155 are widely distributed on hematopoietic, epithelial, and endothelial cells as well as on several tumors [22]. Understanding the interaction between tumors and the immune system has helped in the initiation of different immune-mediated therapies to improve patient management. Moreover, NK cell based therapy leads to stabilization of disease with mild side effects [23]. Ovarian cancer incidence rate, highest mortality rate, availability of treatment options indicate the importance of bridging studies from India to rationalize alternate treatment with correct prognosis. Hence, we proposed to study the differential immune profile of women with primary and recurrent ovarian cancer. The study focused on the frequency, the phenotype of NK, NKT-like, and T cells, their cognate ligands as well as cytokine profiles, in clinical samples such as blood and tissue to highlight differential immune profiles and their prognostic significance.

## Material and methods

### Participant enrollment

Primary and recurrent epithelial ovarian cancer (EOC) patients, who underwent surgical resection at Saifee Hospital and P.D. Hinduja Hospital & Medical Research Center, Mumbai, India between 2017 and 2021, were enrolled in the study. Patients who were initially diagnosed with epithelial ovarian cancer and visiting the hospitals because of relapse of the diseases. These patients without any kind of therapeutic intervention in last 6 months were enrolled as recurrent epithelial ovarian cancer cases. Each enrolled patient signed the informed consent form approved by the Institutional ethics review committee of ICMR-National Institute for Research in Reproductive and Child Health, Mumbai, and both the collaborative hospitals for collection of blood and tissue specimens. Blood and tissue specimens were collected from these cases at the time of surgery in EDTA vacutainer, and DMEM medium respectively to study the immune-phenotype of NK, NKT-like, and T cells. Blood samples from age-matched healthy controls (HC), without any evidence of disease or infection, were collected for comparative analysis. Serum specimens were used to measure cytokine profiles and soluble ligands using ELISAs. Patients with non - serous epithelial ovarian cancer, suffering with other tumor, infection or immune disease were excluded from the study.

### Preparation of single cell suspension from tissue specimens

Tissue specimens were processed by enzymatic and mechanical digestion to get single-cell suspension before staining. Briefly, tissue samples were washed twice with PBS and cut into small pieces, treated with collagenase (4 ml) and incubated at 37 ºC in a water bath for 15 min. Samples were centrifuged at 3000 rpm for 10 min to pellet the cells. Trypsin EDTA (0.05%) was added to the pallet and incubated at 37 ºC for 10 min. Double the volume of 10% FBS in DMEM/PBS (4 ml) was added to the sample tube, centrifuged at 3000 rpm for 10 min to pellet the cells. Pellet was resuspended in DMEM and strained through the cell strainer (40 μm). The single-cell suspension obtained was used for immune-phenotyping of receptors and their cognate ligands [24, 25].

### Immune staining of blood, tissue specimens using flow cytometry

Whole blood (150 μl) and single cell suspension obtained from tissue specimens was stained with fluorescently labeled monoclonal antibodies (Details of antibodies used: supplementary Table S1) and incubated for 30 min at 4ºC in dark. Stained blood samples were incubated in FACS lysis buffer (BD Biosciences) for 15 min with intermittent vortexing and washed twice with staining buffer (0.02% FBS in PBS). A similar staining method as mentioned for blood was used for tissue infiltrating immune cells and surface ligands on tumor cells except the RBC lysis was not required in tissue specimens [24, 25]. Samples were acquired immediately after staining on the BD FACS Aria™ Fusion (BD Biosciences) flow cytometer. The data was analyzed using FlowJo software version 10.1. The threshold for positive staining was determined using unstained or fluorescence minus one (FMO) control. LIVE/DEAD™ Fixable Violet Dead Cell Stain kit (Invitrogen, Vienna, Austria) was used to exclude dead cells. The lymphocyte population was gated based on the CD45 expression for both blood and tissue. To identify circulating and tumor-infiltrating NK, NKT-like, and T cells a sequential gating strategy was created based on the CD3 and CD56 expression. NK cells were further divided into bright NK (CD56^Bright^ NK) and Dim NK (CD56^Dim^ NK) cells based on the density of CD56 expression. The expression of phenotypic markers on circulating and tumor-infiltrating CD56^Bright^ NK, CD56^Dim^ NK, NKT-like, and T cells was then evaluated by the percentage of positive cells (Supplementary Fig. S1). For ligand panels after exclusion of dead cells, a singlet gate was used to remove the debris and doublets followed by gating for individual ligands (Supplementary Fig. S2).

### Procartaplex multiplex immunoassay

Soluble serum level of cytokines such as IL-2, IL-5, IL-6, IL-8, IL-10, IL-15, IL-27, IFN-У, TNF-α, GM-CSF, and two soluble ligands B7-H6, Poliovirus receptor (PVR) was determined by using Procartaplex Multiplex Immunoassay (Invitrogen, Vienna, Austria). The protocol was followed according to the manufacturer’s instructions. All the reagents, plastic wares used in the process were supplied with the kit. Briefly, the antibody coated beads against the different cytokines or ligands of interest were processed in a 96-well plate. A flat magnet ELISA plate holder was used for the whole process. The beads were washed with wash buffer for 30 seconds, followed by incubation with serum and standards for 1 hour. This was followed by washings and addition of enzyme linked secondary antibodies against all these cytokines and ligands of interest. After incubation, the plate containing the beads was washed. This was followed by addition of streptavidin–R-phycoerythrin (SAPE) to capture the enzyme linked antigen-antibody complex. After 30 min incubation the plate was washed. The captured antigen–antibody complex beads present in the plate was analyzed on a Luminex™ instrument (Thermo Fisher Scientific, USA) to measure their concentration.

### Soluble serum level of MICA, MICB, and ULBP-1 analyzed by ELISA

All the ELISAs were done according to the manufacturer’s instructions (Invitrogen, Vienna, Austria). Absorbance was taken on a spectrophotometer using 450 nm as the primary wavelength. Standards of known concentrations, provided in the kit were used to plot the standard curve, against which the OD of each sample was used to get the concentration of these ligands.

### Statistical analysis

GraphPad Prism 9.0 (GraphPad Software, San Diego, CA, USA) was used for all statistical analysis. Data was presented as a scatter dot plot. Comparison between the groups was drawn using an unpaired Mann-Whitney U test. All data are reported as means ± SEM (standard error of mean). Linear regression and spearman’s correlation coefficient were used to determine the association between variables. Comparison between paired blood and tissue specimens was drawn using Wilcoxon matched paired signed rank test. *P* values < 0.05 were considered to be statistically significant.

## Results

### Patient’s characteristics

Among the enrolled 51 EOC patients, 33 were primary high grade serous epithelial ovarian cancer (pEOC) and 18 were recurrent epithelial ovarian cancer (rEOC) patients. Blood and tissue specimens from primary epithelial ovarian cancer were collected at the time of primary debulking surgery without any prior intervention. Whereas, tissue and blood samples were collected from those patients initially diagnosed with epithelial ovarian cancer and visiting the hospitals because of relapse of the disease and those who did not underwent any kind of therapeutic intervention in last 6 months were enrolled in recurrent group. The median age of enrolled pEOC patients was 51.5 (Range 34–74) years and for rEOC median age was 50 (Range 40–66) years. Majority of these high grade serous epithelial ovarian cancer patients were at stage III and IV of the disease. Detailed characteristics of both the EOC groups are shown in (Table 1). Blood samples from 46 age-matched HC (Median age: 51, range: 30–64 years) were used for comparative analysis.

**Table 1:** Demographic and clinicopathological details of EOC patients (*n* = 53)

Patients	Characterstics	
	Primary EOC (*n* = 33)	Recurrent EOC (*n* = 18)
Age Median (Range)	51.5 (34–74)	50 (40–66)
**Stage**		
I	0 (0.0 %)	0 (0.0%)
II	4 (12.1%)	0 (0.0%)
III	21 (63.6 %)	11 (61.6%)
IV	8 (24.2 % )	6 (33.3%)
Unknown	0 ( 0.0 %)	1 (5.5%)
**Grade**		
High	28 (84.8%)	15 (83.3%)
Low	2 (6.0%)	1 (5.5%)
Unknown	3 (9.0%)	2 (11.1%)
**Co-morbidity**		
Yes	17 (51.5%)	6 (33.3%)
No	13 (39.3%)	11 (61.1%)
Unknown	3 (0.9%)	1 (5.5%)
**ASC+ Tumor**		
Positive	18 (54.5%)	3 (16.6%)
Negative	11 (33.3%)	14 (77.7%)
Unknown	4 (12.1%)	1 (5.5%)
**Lymph node metastasis**		
Positive	18 (54.5%)	10 (55.5%)
Negative	13 (39.3%)	7 (38.8%)
Unknown	2 (6.0%)	1 (5.5%)
**CA-125 Median (Range)**		
Pretreatment	503.75 (68.5–5111)	241.7 (80–1320)
Post treatment	14.7 (3.9–293.7)	43.35 (8.582–238.9)

*Abbreviations*: *EOC* Epithelial Ovarian Cancer, *ASC+ tumor* Ascitic fluid positive for tumor cells, *CA-125* Cancer Antigen - 125

### Frequency of CD56^Bright^NK, CD56^Dim^NK, NKT-like, and T cells of EOC patients

**Fig. 1:**
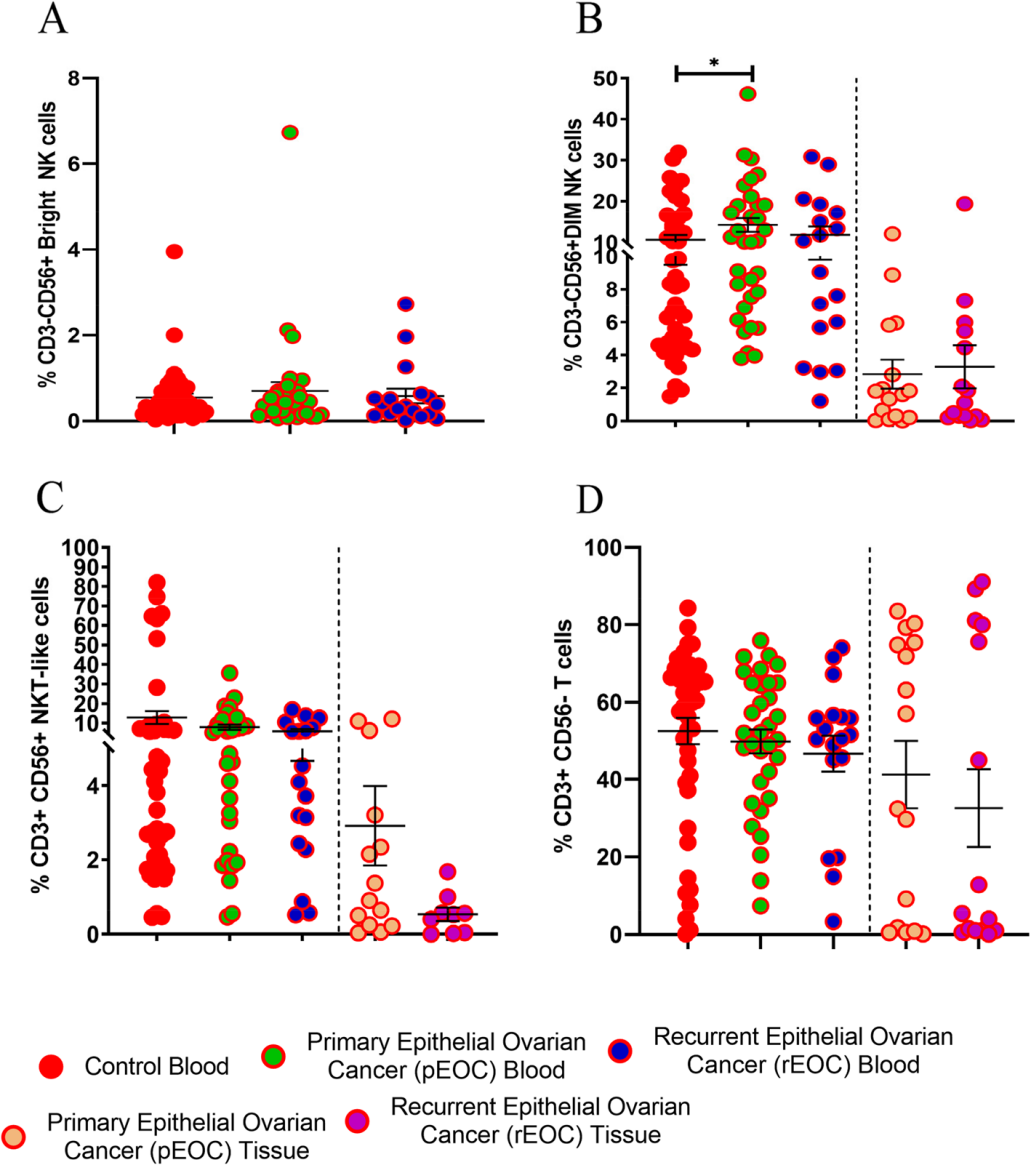
Frequency of immune cell subsets; **A**) CD56^Bright^NK, **B**) CD56^Dim^ NK, **C**) NKT-like, **D**) T Cells in healthy control, pEOC and rEOC patients. *p < 0.05

Comparative analysis on the frequency of CD56^Bright^NK, CD56^Dim^NK, NKT-like, and T cells between peripheral blood of EOC patients and control was carried out (Fig. 1). Within the total lymphocyte fraction, circulatory CD56^Dim^ NK cells were significantly increased in pEOC (*p* = 0.0483) patients (Fig. 1B). However, the percentage of ciculatory CD56^Bright^ NK cells, NKT-like and T cells in both groups of EOC patients were quite comparable with the healthy controls. Similarly, frequency of tumor infiltrated immune cells was also comparable between tumor specimens of both groups of patients (Fig. 1B, C, D). Tumor infiltrated NK cells had low density of CD56 expression based on median fluorescence intensity (MFI).

### Surface receptor expression on CD56^Bright^ NK in peripheral blood of EOC patients

**Fig. 2:**
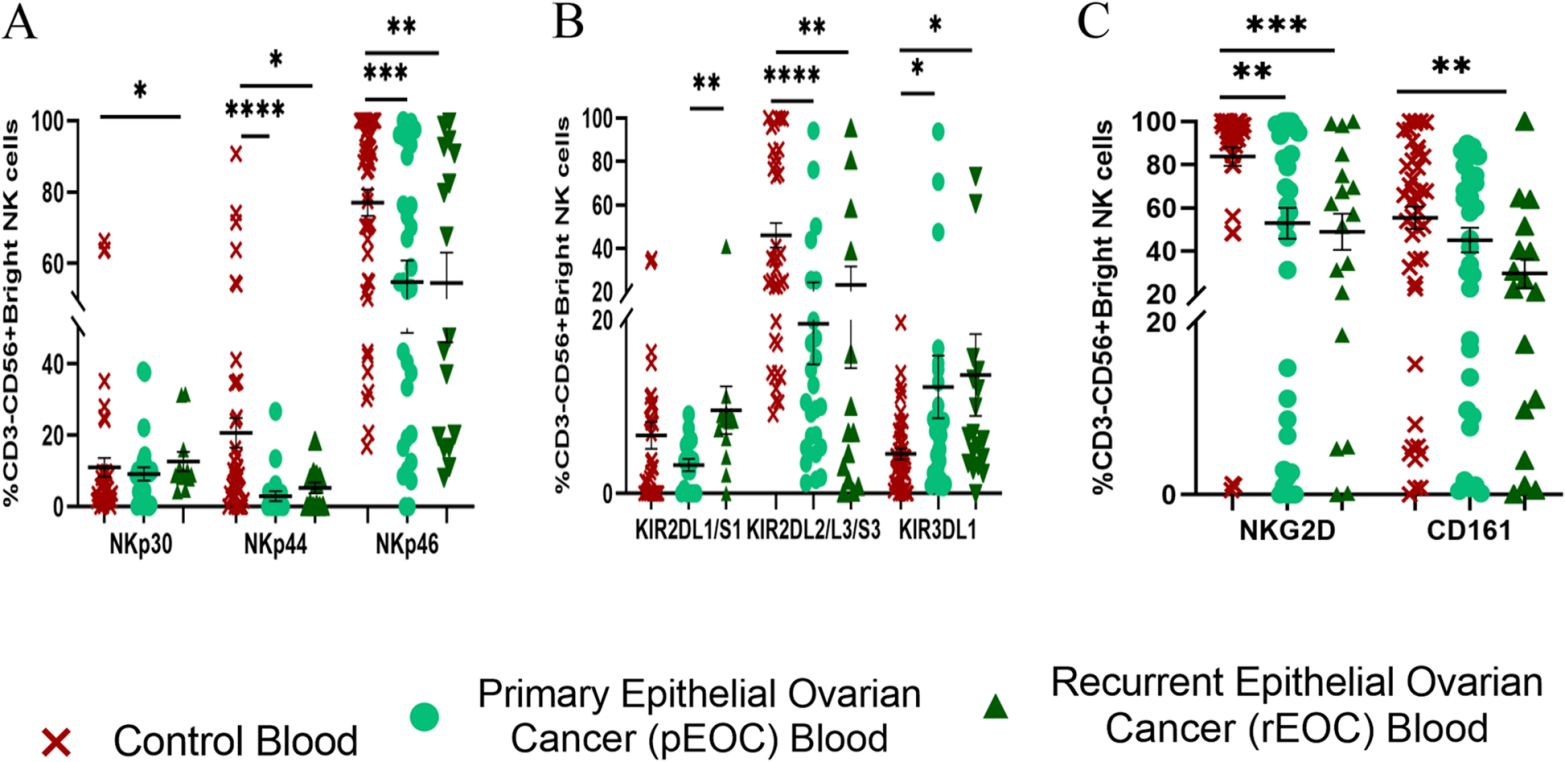
Receptor expression profile of CD56^Bright^ NK cell (**A**-**C**); **A**) NCR group of receptors. **B**) KIR group of receptors. **C**) Expression of NKG2D and CD161 in healthy control, pEOC and rEOC patients. *p < 0.05; **p < 0.01; ***p < 0.001; ****p < 0.0001

Immune dysregulation was observed in the expression of NCR, KIR, and NKG2 group of receptors. Expression of various NK cell receptors was compared between the controls, pEOC and rEOC patients. NKp30 + CD56^Bright^NK were increased in rEOC group while NKp44 + CD56^Bright^NK cells, NKp46 + CD56^Bright^NK cells were decreased in both groups of EOC (Fig. 2A). KIR2DL1/S1 + CD56^Bright^NK cells were increased in rEOC group whereas KIR2DL2/L3/S3 + CD56^Bright^NK cells were decreased in both groups of EOC. In contrast KIR3DL1 + CD56^Bright^NK cells were increased significantly in both the groups when compared with that of HC (Fig. 2B). NKG2D + CD56^Bright^NK cells were reduced in both EOC groups while CD161 + CD56^Bright^NK cells were decreased significantly in rEOC when compared with that of HC (Fig. 2C). Thus, NKp30, CD161 and KIR2DL1/S1 were differentially expressed in the rEOC group of patients whereas prominent activating receptors NKp44, NKp46 and NKG2D were reduced in both groups of EOC patients.

### Surface receptor expression on CD56^Dim^ NK cells of EOC patients

**Fig. 3:**
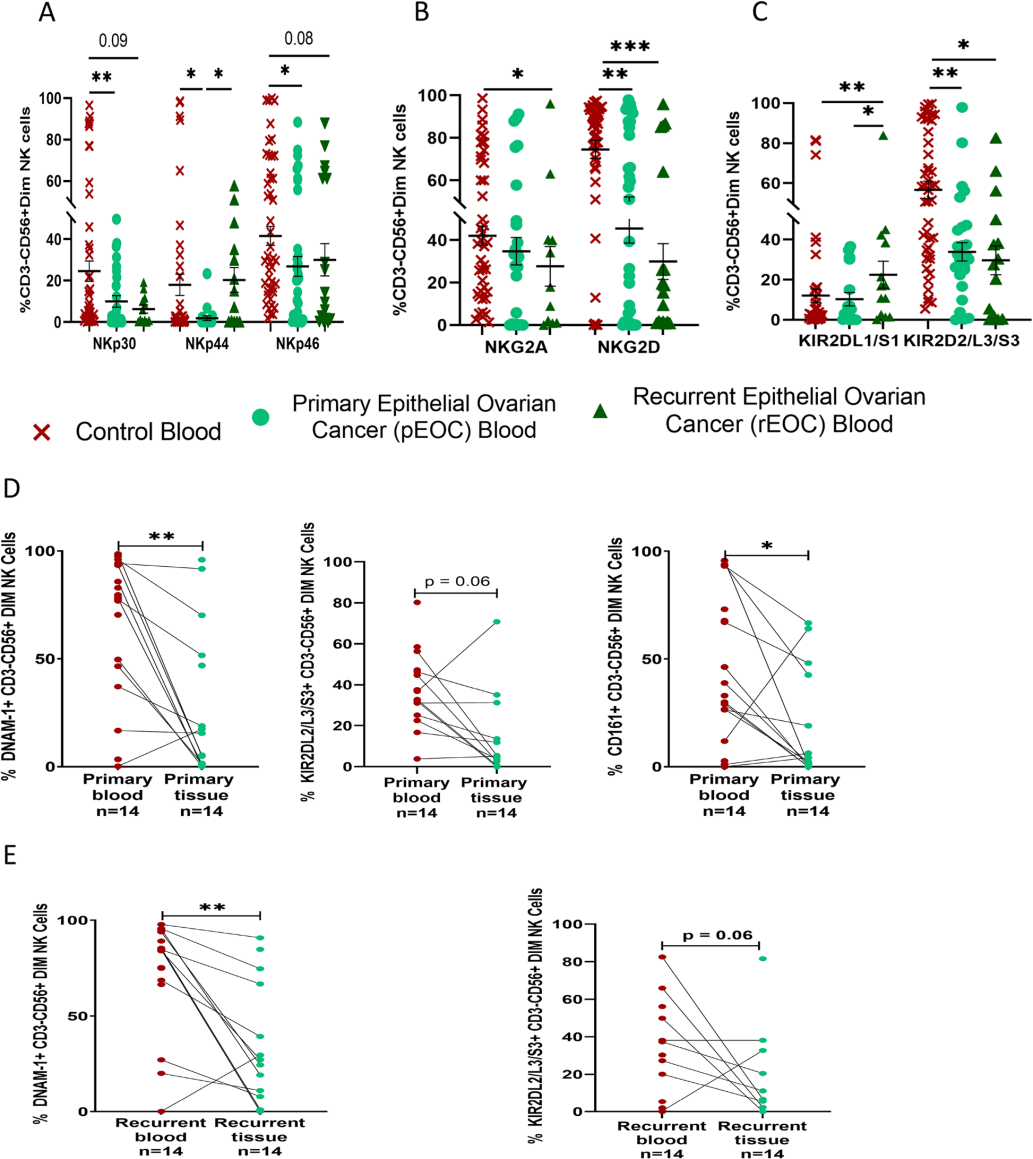
Receptor expression profile of circulatory CD56^Dim^NK cell (**A**-**C**); **A**) NCR group of receptors. **B**) NKG2 group of receptors. **C**) KIR group of receptors in healthy control, pEOC and rEOC patients. Wilcoxon paired analysis (**D**-**E**); between circulatory and tumor infiltrated immune cells in **D**) pEOC patients and **E**) rEOC patients. *p < 0.05; **p < 0.01; ***p < 0.001

Evaluation of peripheral blood of both group of patients revealed apparent changes in the phenotype of CD56^Dim^NK cells with significant down-regulation of activating NCR receptors in pEOC patients. Circulatory NKp30 + CD56^Dim^NK, NKp44 + CD56^Dim^NK, NKp46 + CD56^Dim^NK cells were reduced significantly in pEOC when compared with HC. There was a trend of increased circulatory NKp44 + CD56^Dim^NK cells in rEOC than their level in pEOC group (Fig. 3A). Interestingly, NKG2A + CD56^Dim^NK cells were reduced significantly in rEOC patients. Circulatory NKG2D + CD56^Dim^NK, cells were significantly reduced in both groups of patients when compared with HC (Fig. 3B). KIR2DL1/S1 + CD56^Dim^NK cells were increased in rEOC when compared with pEOC and HC whereas KIR2DL2/L3/S3 + CD56^Dim^NK cells were reduced in both group of patients (Fig. 3C). No significant difference in receptor expression profile was found on tumor infiltrated CD56^Dim^NK cells between both groups of patients (Supplementary Fig. S3a). Wilcoxon paired analysis revealed that tumor infiltrated DNAM-1 + CD56^Dim^NK cells were reduced in both groups than their circulatory counterpart. Furthermore, tumor infiltrated CD161 + CD56^Dim^NK cells were reduced in pEOC than their circulatory counterparts (Fig. 3D, E). In Summary, activated NKp44 + CD56^Dim^ NK cells and KIR2DL1/S1 + CD56^Dim^ NK cells were increased specifically in rEOC.

### Surface receptor expression on NKT-like cells of EOC patients

**Fig. 4:**
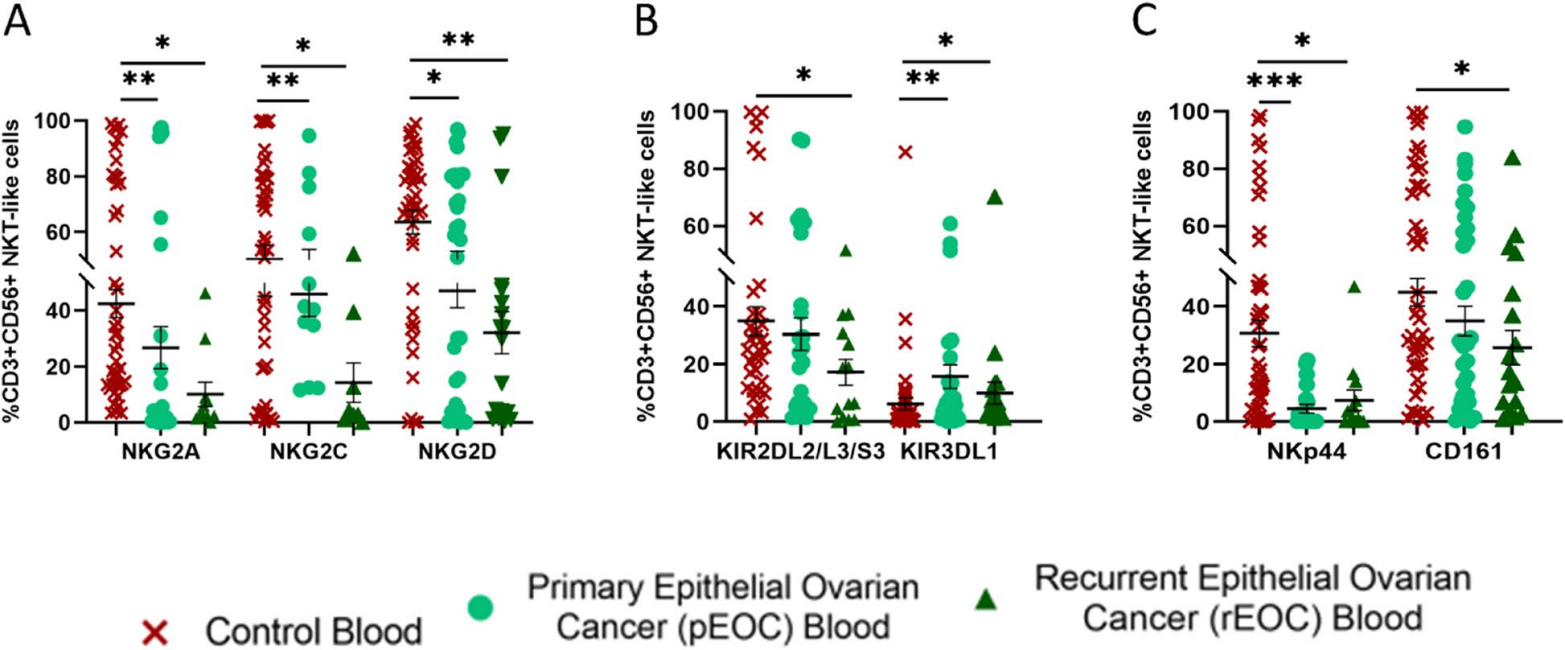
Receptor expression profile of circulatory CD3 + CD56 + NKT-like cell (**A**-**C**); **A**) NKG2 group of receptors. **B**) KIR group of receptors. **C**) Expression of NKp44 and CD161 in healthy control, pEOC and rEOC patients *p < 0.05; **p < 0.01; ***p < 0.001

Among NKG2 group of receptors circulatory NKG2A + NKT-like, NKG2C + NKT-like, NKG2D + NKT-like cells were reduced in both groups than HC (Fig. 4A). Among KIR group, circulatory KIR2DL2/L3/S3 + NKT-like cells were reduced only in rEOC. In contrast, circulatory KIR3DL1 + NKT-like cells were increased significantly in both groups than HC (Fig. 4B). Analysis of the surface expression of these receptors on circulating NKT-like cells revealed that circulatory NKp44 + NKT-like cells were decreased significantly in both groups, whereas circulatory CD161 + NKT-like cells were decreased in rEOC than HC (Fig. 4C). No significant difference was seen in tumor infiltrated NKT-like cells between the groups of these EOC patients (Supplementary Fig. S3B). Unlike NK cells, complete NKG2 group of receptors were reduced on NKT-like cells in both groups of patients. NKT-like cells with CD161, KIR2DL2/L3/S3 were reduced specifically in rEOC patients.

### Surface receptor expression on T cells of EOC patients

**Fig. 5:**
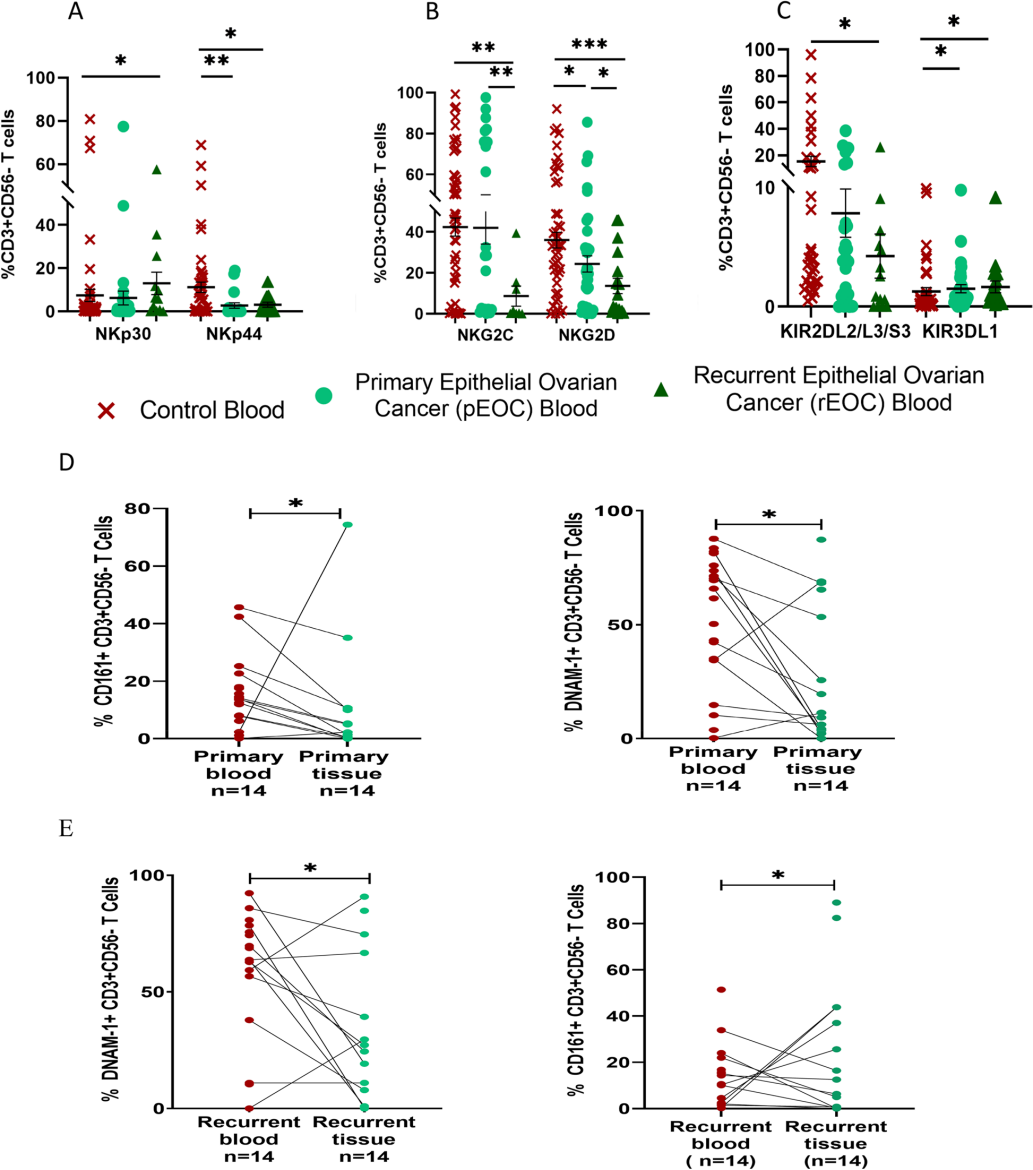
Receptor expression profile of circulatory CD3 + CD56-T cells (**A**-**C**); **A**) NCR group of receptors. **B**) NKG2 group of receptors. **C**) KIR group of receptors in healthy control, pEOC and rEOC patients. Wilcoxon paired analysis (**D**-**E**); between tumor infiltrated and circulatory T cells in **D**) pEOC patients and **E**) rEOC patients. *p < 0.05; **p < 0.01; ***p < 0.001

Assessment of these receptors on T cells indicates the deregulation of receptor expression profile on T cells in both groups of EOC patients. Circulatory NKp30 + T cells were increased significantly in rEOC, while NKp44 + T cells were decreased significantly in both groups when compared with HC (Fig. 5A). Among NKG2 group, circulatory NKG2C + T cells were reduced in rEOC than pEOC and HC. Whereas, NKG2D + T cells were reduced significantly in both groups when compared with HC, reduction was more prominent in rEOC (Fig. 5B). In KIR group, KIR2DL2/L3/S3 + T cells were reduced significantly in rEOC, while KIR3DL1 + T cells were increased in both groups (Fig. 5C). Furthermore, expression of these receptors has no significant difference on tumor infiltrated T cells between both groups of patients (Supplementary Fig. S3C). However, paired Wilcoxon analysis revealed decreased level of DNAM-1 + T cells and CD161 + T cells in both groups than their circulatory counterparts (Fig. 5D, E).

### Surface and soluble ligand levels in EOC patients

**Fig. 6:**
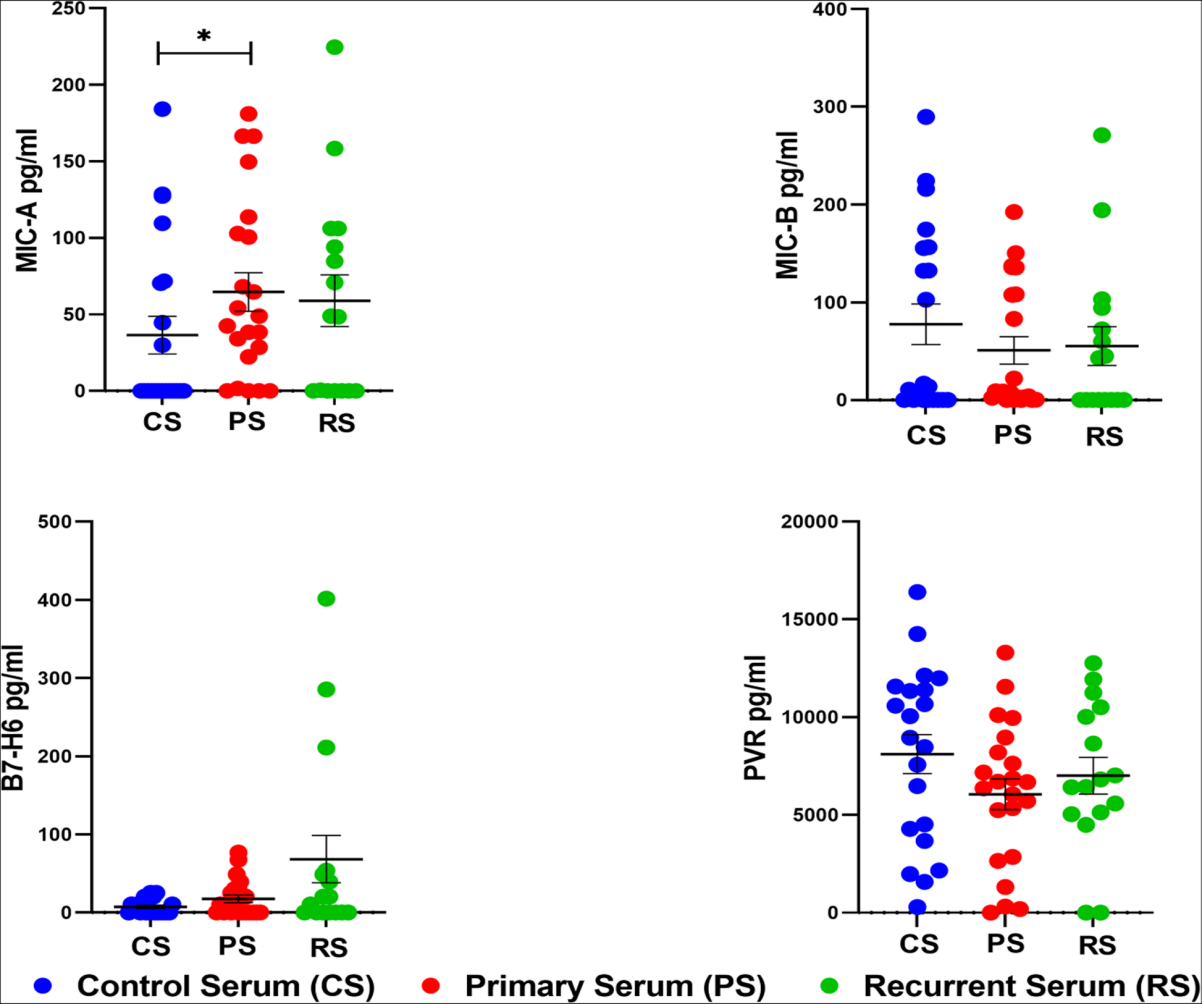
Serum level of soluble ligands of healthy control, primary and recurrent EOC patients *p < 0.05

The surface level of MICA, MICB, ULBP-1, HLA-E, B7-H6, LLT-1, PVR, and VIMENTIN on tumor cells were comparable between both groups (Supplementary Fig. S4). Measurement of serum level of MICB, B7-H6, and PVR did not show any significant difference in both groups of patients when compared with the HC except for MICA which was high in both the groups, significantly increased in pEOC compared to HC (Fig. 6).

### Serum cytokine profile of EOC patients

**Fig. 7:**
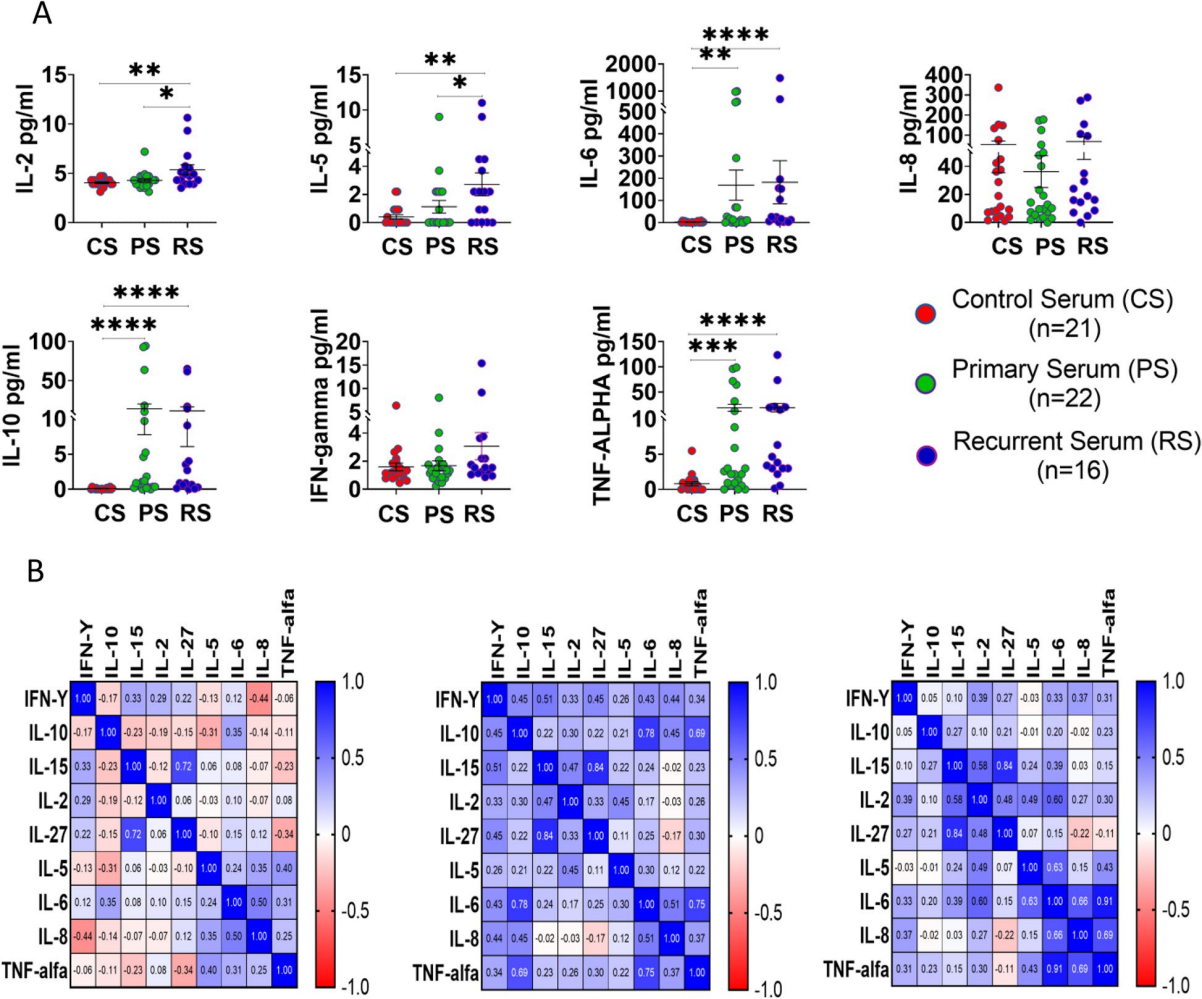
**A**) Serum cytokine level in healthy control, primary and recurrent EOC patients and **B**) Spearman correlation matrix of cytokine panel in healthy control, primary and recurrent EOC patients *p < 0.05; **p < 0.01; ***p < 0.001; ****p < 0.0001

A panel of cytokines [IL-2, IL-5, IL-6, IL-8, IL-10, IL-15, IL-27, IFN-У, TNF-α, GM-CSF and two soluble ligands B7-H6, Poliovirus receptor (PVR)] were measured by multiplex beads immunoassay in the sera of 21 HC, 22 pEOC, and 16 rEOC patients. The serum level of IL-2 and IL-5 were significantly increased in rEOC than pEOC and HC. Moreover, IL-6, IL10 and TNF-α were significantly elevated in both groups of patients (Fig. 7A). Spearman correlation matrix further confirms the disrupted cytokine correlation in both groups of ovarian cancer patients (Fig. 7B).

### Correlation between cytokine level and the receptor expression profile of NK, NKT-like, and T cells in EOC patients

Cytokines widely regulate the function of the immune system, inflammation, and homeostasis, so we analyzed spearman’s correlation between cytokine level and the receptor expression profile of NK cell subsets, NKT-like and T cells, which indicated disruption of the balance between cytokines and receptor expression of these cells in both groups of EOC patients. In the given Supplementary Table S2, we have reported the significant correlation, either positively or negatively among these two groups of patients as well as in HC. Among the analyzed cytokines, only IL-2, IL-5, IL-6, IL-8, IL-10, IFN-γ, and TNF-α were significantly correlated with the expression of receptors on these immune cells. IL-8 levels were negatively correlated with different receptors of HC, such as NKp30 + CD56^Bright^NK, NKG2A + CD56^Bright^NK, DNAM-1 + CD56^Bright^NK, KIR3DL1 + NKT-like and CD161 + T cells. However, IL-8 levels were positively correlated with NKp44 + CD56^Bright^NK, NKG2D + CD56^Bright^NK, NKG2D + CD56^Dim^NK, DNAM-1 + CD56^Dim^NK, NKG2D + NKT-like cells, and CD161 + NKT-like cells of pEOC. Only CD161 + NKT-like cells were negatively correlated with IL-8 in rEOC. IL-2 levels were positively correlated with receptors of HC such as NKp44 + CD56^Bright^NK, NKG2A + CD56^Bright^NK, NKG2D + CD56^Bright^NK, CD161 + CD56^Bright^NK cells and KIR3DL1 + CD56^Dim^NK cells. In HC, only CD161 + T cells were negatively correlated with IL-2 levels. However, no such type of correlation was seen between these receptors and IL-2 in both groups of EOC patients. In HC, IL-6 was negatively correlated with NKG2A + CD56^Bright^NK, DNAM-1 + CD56^Bright^NK, DNAM-1 + CD56^Dim^NK, CD161 + T and KIR2DL2/L3/S3 + T cells. However, in pEOC, IL-6 level was positively correlated with NKp46 + CD56^Bright^NK and KIR3DL1 + T cells. IL-10 was negatively correlated with NKG2D + CD56^Bright^NK, CD161 + CD56^Bright^NK of HC as well as with CD161 + T cells of rEOC. In pEOC only NKp46 + CD56^Bright^NK cells were positively correlated with IL-10 levels. IL-5 level was only positively correlated with KIR2DL2/L3/S3 + CD56^Dim^NK cells of HC while IL-5 does not correlate with any of the receptors in EOC patients. In HC, IFN-γ levels were positively correlated with CD161 + CD56^Bright^NK and NKG2A + CD56^Dim^NK cells, while negatively correlated with NKG2A + T cells. In pEOC, NKp46 + CD56^Bright^NK cells were positively correlated with IFN-γ levels. TNF-α levels were negatively correlated with NKG2C + CD56^Dim^NK, KIR2DL2/L3/S3 + CD56^Dim^ NK, DNAM-1 + CD56^Dim^NK cells of HC (Supplementary Table S2).

## Discussion

This is the first study to the best of our knowledge, to report expression of NK cell receptors, their surface ligands, soluble ligands and cytokine profile in Indian women with primary or recurrent ovarian cancer. Immune escape is one of the most challenging questions in cancer biology and how it affects the development of solid tumors remains unanswered. Antitumor immunity was found to be compromised in solid tumors [26, 27]. We have found immune changes in pEOC patients in terms of increased frequency of circulating CD56^Dim^NK cells which may indicate proliferation and activation of innate immune cells. A high level of circulatory NK cells in colorectal cancer was associated with better survival and can be used as an independent prognosticator [28]. However, other circulatory subsets such as CD56^Bright^NK, NKT-like, and T cells were comparable with HC in both groups of EOC. The modulation of receptor expression could be an immune escape mechanism in invasive pEOC and rEOC. We have found that alteration of these receptors was more drastic on circulatory immune cells. Two novel mechanisms for down regulation of receptors on circulatory immune cells are the release of soluble ligands as “decoy molecules” which suppresses the expression of target receptors and the release of platelet derived TGF-β which down-regulates the expression of DNAM-1 and CD96 [29]. Present analysis on these soluble ligands revealed, high soluble MICA level in both groups of cases. Increased soluble MICA may lead to consistent decrease of NKG2D + immune cells. Modulation of surface receptor expression has been reported in several other malignancies [30, 31]. Down-regulation of activating receptors NKp30, NKp46, and NKG2D is an immune evasion mechanism which led to low cytolytic activity [27]. In our data, both subsets of NK cells with activating phenotype were reduced which might have lead to immune suppression. Moreover, we also noticed the change in the expression pattern of inhibitory receptors on NK cells in both groups of EOC patients. Circulatory immune cell subsets such as NKp46 + CD56^Bright^NK, NKp30 + CD56^Dim^NK, NKp46 + CD56^Dim^NK, NKp44 + NKT-like, NKG2A + NKT-like, and NKG2A + T cells were reduced in both groups of EOC patients. This probably indicates immune dysregulation and common receptor expression pattern at primary and recurrent stage of the disease. However, certain subsets such as NKp30 + CD56^Bright^NK, NKp44 + T and NKp46 + T cells were reduced specifically in pEOC. In contrast, the frequencies of NKp44 + CD56^Dim^NK cells were increased, whereas CD161 + CD56^Bright^NK, NKG2A + CD56^Dim^NK, CD161 + NKT-like, and NKp30 + T cells were reduced in rEOC, depicting the immunological differences among these two groups of patients. Clinically relevant anti-metastatic role of NK cells was reported in several solid tumors [32, 33]. Thus, reduction in circulatory subsets with activating phenotype could have facilitated the breach of local tumor microenvironment by neoplastic cells, reach the circulation and colonize at distant sites. Furthermore, differential reduction in specific subsets, especially in recurrent cancer may be used to rationalize alternative therapeutic strategies and special care of relapsed cases. Again, these differential immune profiles might be a factor of time. As the disease progresses, the immune signature of pEOC undergoes some changes in circulation that might facilitate the disease relapse. Further, immune infiltration has been neglected for a long time but the tumor infiltrated immune cells can also predict the outcome of ovarian cancer [34]. The tumor infiltrated NK and T cell subsets positive for DNAM-1 are reduced in both groups of patients. Reduction in DNAM-1 receptor especially on NK cells in TME might have led to decrease in NK cell’s ability of synapse formation, a key step to execute the effecter function. Tumor-infiltrating T cells demonstrated a beneficial effect on ovarian cancer patients, especially as a predictive biomarker for the prognosis of ovarian cancer patients suggesting that these cells also play a major role in the outcome of the disease in EOC [23]. Profile of both groups of tumors was equally immunogenic in terms of ligand expression by tumor cells. The expression of ligands by tumor cells can modulate the phenotype of circulatory immune cells [35]. Further, we evaluated the serum cytokine level of both groups; exposure to these cytokines can modulate the phenotype and function of immune cells [36]. IL-6 and oncostatin M have been found to directly stimulate enhanced invasion of cancer cells, stimulate the promotion of cell cycle, enhance resistance to chemotherapy, and cause epithelial-to-mesenchymal transition (EMT) [37]. IL-10 was also associated with ovarian cancer cell migration and the worst disease-free survival of ovarian cancer patients [38]. Furthermore, TNF-α and IL-6 induces the generation of reactive oxygen species, nitrogen species and promote DNA damage which accelerates the initiation of tumorigenesis [39]. Thus, elevated level of IL-6, IL-10 and TNF-α seen in these cases might have led to enhanced invasion, resistance to chemotherapy and disease prognosis. Besides, elevated level of IL-5 and IL-15 in rEOC, it is very interesting to find the elevated level of IL-2 in their sera as IL-2 has promising therapeutic potential, a phase II clinical trial has shown its therapeutic benefits in platinum-resistant ovarian cancer patients [40]. IL-15 also acts as a super agonist that enhances NK cell function against ovarian cancer [41]. However, these beneficial effects of IL-2 and IL-15 are not seen in present study. So, to evaluate the influence of increased cytokine levels on the phenotype of different immune cells, spearman’s correlation analysis was carried out between serum cytokine level and receptor expression profile. Among the elevated cytokines, IL-6 was the only cytokine that was positively correlated with NKp46 + CD56^Bright^NK cells. Similar cytokine profiles in serum of both patient groups were rarely correlated with phenotype of immune cells, while the correlation was more common in healthy control which indicates the disrupted axis of cytokine and immune cell interactions in both the group of cases in spite of different state of the disease. Although, study has its limitation as in our data, we did not have ligand and receptor expression profile from healthy tissue. Another limitation for the study is the small sample size in this multiparametric study. However, the study has its own strength, possibly highlighting for the first time a broad immune profile comprising expression of the surface receptors, ligands, soluble ligands and cytokine profiles in both pEOC and rEOC patients in an Indian scenario.

## Conclusions

The present study highlights the immunological similarity as well as the differences of immune subsets in pEOC and rEOC. Circulatory NKG2D positive NK, NKT-like, and T cells were significantly reduced in both groups of EOC which is the prominent activating marker. A high level of soluble MICA might have acted as a “decoy” molecule. This could be a probable mechanism of decrease in NKG2D positive subsets in both groups of EOC. Furthermore, the study highlights the elevated level of serum cytokines in EOC patients that may help to understand the cytokines associated with ovarian cancer progression. Profiling of tumor infiltrated immune cells revealed the reduced level of DNAM-1 positive NK and T cells in both groups of EOC. A reduced level of DNAM-1 expression on tumor infiltrated NK cells may led to a possible decrease in NK cell’s ability to synapse formation, required to execute the effecter function. Significantly reduced expression of NKG2D, high level of MICA as well as IL-6, IL10 and TNF-α indicates immune suppression of ovarian cancer patients.. It also emphasized that restoration of cytokines level, NKG2D and DNAM-1on tumor infiltrated immune cells may be targeted to develop specific therapeutic approaches for high-grade serous epithelial ovarian cancer.

## Supplementary Information

**Additional file 1: Supplementary Figure S1.** Representative gating strategy for NK, NKT-like, and T cells receptors.

**Additional file 2: Supplementary Figure S2.** Representative gating strategy for ligands panel.

**Additional file 3: Supplementary Figure 3.** Phenotype of tumor infiltrating (a-c) CD56^Dim^ NK, NKT-like and T cell in pEOC and rEOC patients.

**Additional file 4: Supplementary Figure 4.** Percentage of tumor cells positive for cognate ligands of NK cell receptors in tissue specimens of pEOC and rEOC patients.

**Additional file 5: Supplementary Table S1.** Details of antibodies used to stain NK cells receptors and ligands.

**Additional file 6: Supplementary Table S2.** Spearman’s correlation between serum cytokine and receptor expression profile of CD56^Bright^NK, CD56^Dim^NK, NKT-like, and T cells of healthy control, pEOC and rEOC patients.

## Data Availability

The datasets generated during and/or analyzed during the current study are available from the corresponding author on reasonable request. All the generated data are given here as Figures, Table and supplementary material.

## References

[1] Sung H, Ferlay J, Siegel RL, Laversanne M, Soerjomataram I, Jemal A (2021). Global cancer statistics 2020: GLOBOCAN estimates of incidence and mortality worldwide for 36 cancers in 185 countries. CA Cancer J Clin.

[2] Sung PL, Chang YH, Chao KC, Chuang CM (2014). Global distribution pattern of histological subtypes of epithelial ovarian cancer: a database analysis and systematic review. Gynecol Oncol.

[3] Howlader N, Noone AM, Krapcho M, Miller D, Brest A, Yu M, Ruhl J, Tatalovich Z, Mariotto A, Lewis DR, Chen HS, Feuer EJ, Cronin KA (eds). SEER Cancer Statistics Review, 1975-2018, National Cancer Institute. Bethesda, MD. https://seer.cancer.gov/csr/1975_2018/, based on November 2020 SEER data submission, posted to the SEER web site, April 2021.

[4] Jayson GC, Kohn EC, Kitchener HC, Ledermann JA (2014). Ovarian cancer. Lancet (London, England).

[5] Bridget C, Goode EL, Kalli KR, Knutson KL, DeRycke MS (2013). The immune system in the pathogenesis of ovarian cancer. Crit Rev Immunol.

[6] Wright JD, Chen L, Tergas AI, Patankar S, Burke WM, Hou JY, Neugut AI, Ananth CV, Hershman DL. Trends in relative survival for ovarian cancer from 1975 to 2011.Obstet Gynecol. 2015;125(6):1345-1352. 10.1097/AOG.0000000000000854. 10.1097/AOG.0000000000000854 PMC4484269 26000505

[7] Mitra AK, Zillhardt M, Hua Y, Tiwari P, Murmann AE, Peter ME (2012). MicroRNAs reprogram normal fibroblasts into cancer-associated fibroblasts in ovarian cancer. Cancer Discov.

[8] Moretta L (2010). dim human NK cells dissecting. Blood.

[9] Hoogstad-Van Evert JS, Maas RJ, Van Der Meer J, Cany J, Van Der Steen S, Jansen JH (2018). Peritoneal NK cells are responsive to IL-15 and percentages are correlated with outcome in advanced ovarian cancer patients. Oncotarget.

[10] Greppi M, Tabellini G, Patrizi O, Candiani S, Decensi A, Parolini S (2019). Strengthening the antitumor NK cell function for the treatment of ovarian cancer. Int J Mol Sci.

[11] Chan A, Hong D-L, Atzberger A, Kollnberger S, Filer AD, Buckley CD (2007). CD56bright human NK cells differentiate into CD56dim cells: role of contact with peripheral fibroblasts. J Immunol.

[12] Paul S, Lal G (2017). The molecular mechanism of natural killer cells function and its importance in cancer immunotherapy. Front Immunol.

[13] Kumar BV, Connors TJ, Farber DL (2018). Human T cell development, localization, and function throughout life. Immunity.

[14] Westergaard MCW, Andersen R, Chong C, Kjeldsen JW, Pedersen M, Friese C (2019). Tumour-reactive T cell subsets in the microenvironment of ovarian cancer. Br J Cancer.

[15] Melero I, Rouzaut A, Motz GT, Coukos G. T-cell and NK-cell infiltration into solid tumors: a key limiting factor for efficacious cancer immunotherapy. Cancer Discov. 2014;4(5):522-6. 10.1158/2159-8290.CD-13-0985. 10.1158/2159-8290.CD-13-0985 PMC4142435 24795012

[16] Almeida JS, Casanova JM, Santos-Rosa M, Tarazona R, Solana R, Rodrigues-Santos P (2023). Natural killer T-like cells: immunobiology and role in disease. Int J Mol Sci.

[17] Wang H, Yang D, Xu W, Wang Y, Ruan Z, Zhao T (2008). Tumor-derived soluble MICs impair CD3+CD56+ NKT-like cell cytotoxicity in cancer patients. Immunol Lett.

[18] Kroeger DR, Milne K, Nelson BH (2016). Tumor-infiltrating plasma cells are associated with tertiary lymphoid structures, cytolytic T-cell responses, and superior prognosis in ovarian cancer. Clin Cancer Res.

[19] Konjević G, Miräjaić Martinovi K, Jurišić V, Babović N, Spužić I (2009). Biomarkers of suppressed natural killer (NK) cell function in metastatic melanoma: decreased NKG2D and increased CD158a receptors on CD3-CD16+ NK cells. Biomarkers.

[20] Mamessier E, Sylvain A, Bertucci F, Castellano R, Finetti P, Houvenaeghel G (2011). Human breast tumor cells induce self-tolerance mechanisms to avoid NKG2D-mediated and DNAM-mediated NK cell recognition. Cancer Res.

[21] Spear P, Wu MR, Sentman ML, Sentman CL (2013). Nkg2d ligands as therapeutic targets. Cancer Immun.

[22] Zingoni A, Ardolino M, Santoni A, Cerboni C (2012). NKG2D and DNAM-1 activating receptors and their ligands in NK-T cell interactions: role in the NK cell-mediated negative regulation of T cell responses. Front Immunol.

[23] Hoogstad-van Evert JS, Bekkers R, Ottevanger N, Jansen JH, Massuger L, Dolstra H (2020). Harnessing natural killer cells for the treatment of ovarian cancer. Gynecol Oncol.

[24] Kumar P, Ranmale S, Tongaonkar H, Mania-Pramanik J (2022). Immune profile of blood, tissue and peritoneal fluid: a comparative study in high grade serous epithelial ovarian cancer patients at interval debulking surgery. Vaccines.

[25] Czupalla C, Yousef H, Wyss-Coray T, Butcher E (2018). Collagenase-based single cell isolation of primary murine brain endothelial cells using flow cytometry. Bio-Protoc.

[26] Nieto-Velázquez NG, Torres-Ramos YD, Muñoz-Sánchez JL, Espinosa-Godoy L, Gómez-Cortés S, Moreno J (2016). Altered expression of natural cytotoxicity receptors and NKG2D on peripheral blood NK cell subsets in breast cancer patients. Transl Oncol.

[27] Garcia-Iglesias T, del Toro-Arreola A, Albarran-Somoza B, del Toro-Arreola S, Sanchez-Hernandez PE, Ramirez-Dueñas M (2009). Low NKp30, NKp46 and NKG2D expression and reduced cytotoxic activity on NK cells in cervical cancer and precursor lesions. BMC Cancer.

[28] Tang Y, Xie M, Li K, Li J, Cai Z, Hu B (2020). Prognostic value of peripheral blood natural killer cells in colorectal cancer. BMC Gastroenterol.

[29] Cluxton CD, Spillane C, O’Toole SA, Sheils O, Gardiner CM, O’Leary JJ (2019). Suppression of natural killer cell NKG2D and CD226 anti-tumour cascades by platelet cloaked cancer cells: Implications for the metastatic cascade. PLoS One.

[30] Mariel GC, Edith CTI, Pilar CR, Elena GDN, Humberto RM, Guadalupe MSM (2018). Expression of NK cell surface receptors in breast cancer tissue as predictors of resistance to antineoplastic treatment. Technol Cancer Res Treat.

[31] Stringaris K, Sekine T, Khoder A, Alsuliman A, Razzaghi B, Sargeant R (2014). Leukemia-induced phenotypic and functional defects in natural killer cells predict failure to achieve remission in acute myeloid leukemia. Haematologica.

[32] Pasero C, Gravis G, Guerin M, Granjeaud S, Thomassin-Piana J, Rocchi P (2016). Inherent and tumor-driven immune tolerance in the prostate microenvironment impairs natural killer cell antitumor activity. Cancer Res.

[33] Semeraro M, Rusakiewicz S, Zitvogel L, Kroemer G (2015). Natural killer cell mediated immunosurveillance of pediatric neuroblastoma. Oncoimmunology.

[34] Henriksen JR, Donskov F, Waldstrøm M, Jakobsen A, Hjortkjaer M, Petersen CB (2020). Favorable prognostic impact of natural killer cells and T cells in high-grade serous ovarian carcinoma. Acta Oncol.

[35] Krijgsman D, Roelands J, Andersen MN, Wieringa CHLA, Tollenaar RAEM, Hendrickx W (2020). Expression of NK cell receptor ligands in primary colorectal cancer tissue in relation to the phenotype of circulating NK- and NKT cells, and clinical outcome. Mol Immunol.

[36] Brady J, Carotta S, Thong RPL, Chan CJ, Hayakawa Y, Smyth MJ (2010). The interactions of multiple cytokines control NK cell maturation. J Immunol.

[37] Browning L, Patel MR, Horvath EB, Tawara K, Jorcyk CL (2018). IL-6 and ovarian cancer: inflammatory cytokines in promotion of metastasis. Cancer Manag Res.

[38] Lane D, Matte I, Garde-Granger P, Bessette P, Piché A (2018). Ascites IL-10 promotes ovarian cancer cell migration. Cancer Microenviron.

[39] Amin MN, Siddiqui SA, Ibrahim M, Hakim ML, Ahammed MS, Kabir A (2020). Inflammatory cytokines in the pathogenesis of cardiovascular disease and cancer. SAGE Open Med.

[40] Vlad AM, Budiu RA, Lenzner DE, Wang Y, Thaller JA, Colonello K (2010). A phase II trial of intraperitoneal interleukin-2 in patients with platinum-resistant or platinum-refractory ovarian cancer. Cancer Immunol Immunother.

[41] Felices M, Chu S, Kodal B, Bendzick L, Ryan C, Lenvik AJ (2017). IL-15 super-agonist (ALT-803) enhances natural killer (NK) cell function against ovarian cancer. Gynecol Oncol.

