# Supplementary figures and images for: Immune profile of primary and recurrent epithelial ovarian cancer cases indicates immune suppression, a major cause of progression and relapse of ovarian cancer
Source: J Ovarian Res. 2023 Jun 15;16:114. doi: 10.1186/s13048-023-01192-4 (PMC10268537; doi:10.1186/s13048-023-01192-4)

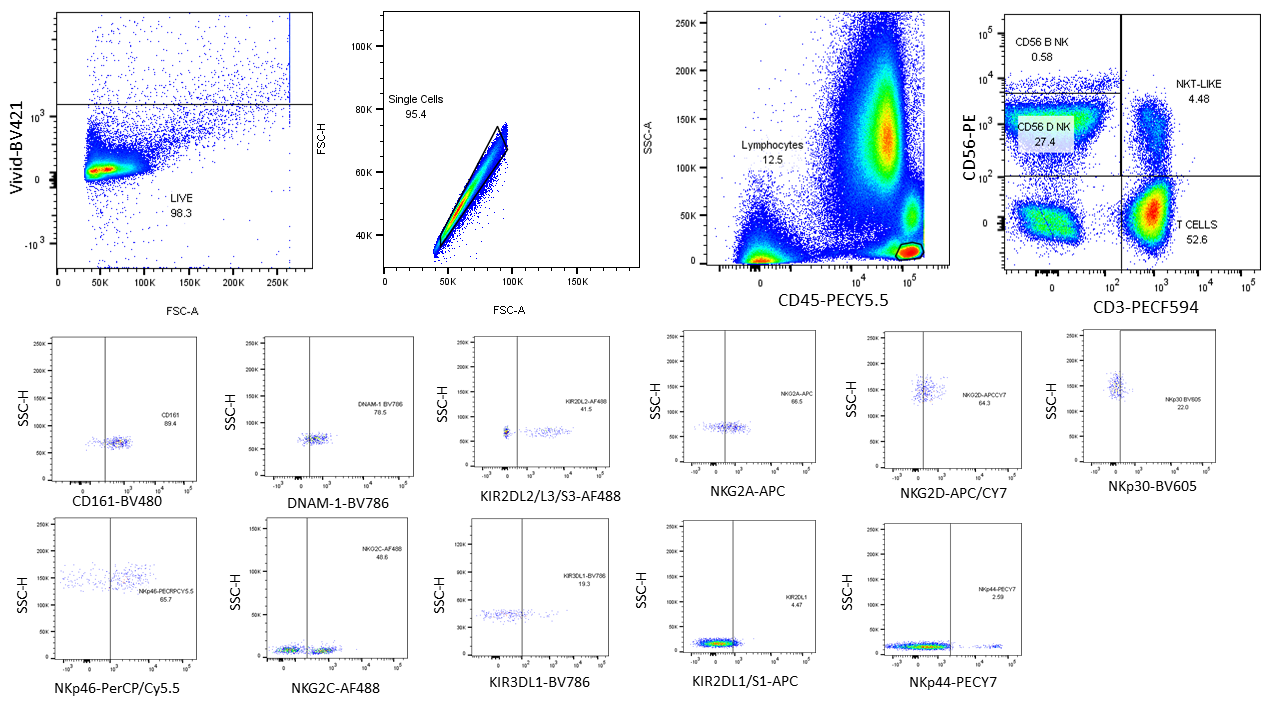


**Supplementary Fig. 1** Representative gating strategy for NK, NKT-like, and T cells receptors

Supplement: Supplementary file 1 — Additional file 1: Supplementary Figure S1. Representative gating strategy for NK, NKT-like, and T cells receptors. [file 13048_2023_1192_MOESM1_ESM.docx]

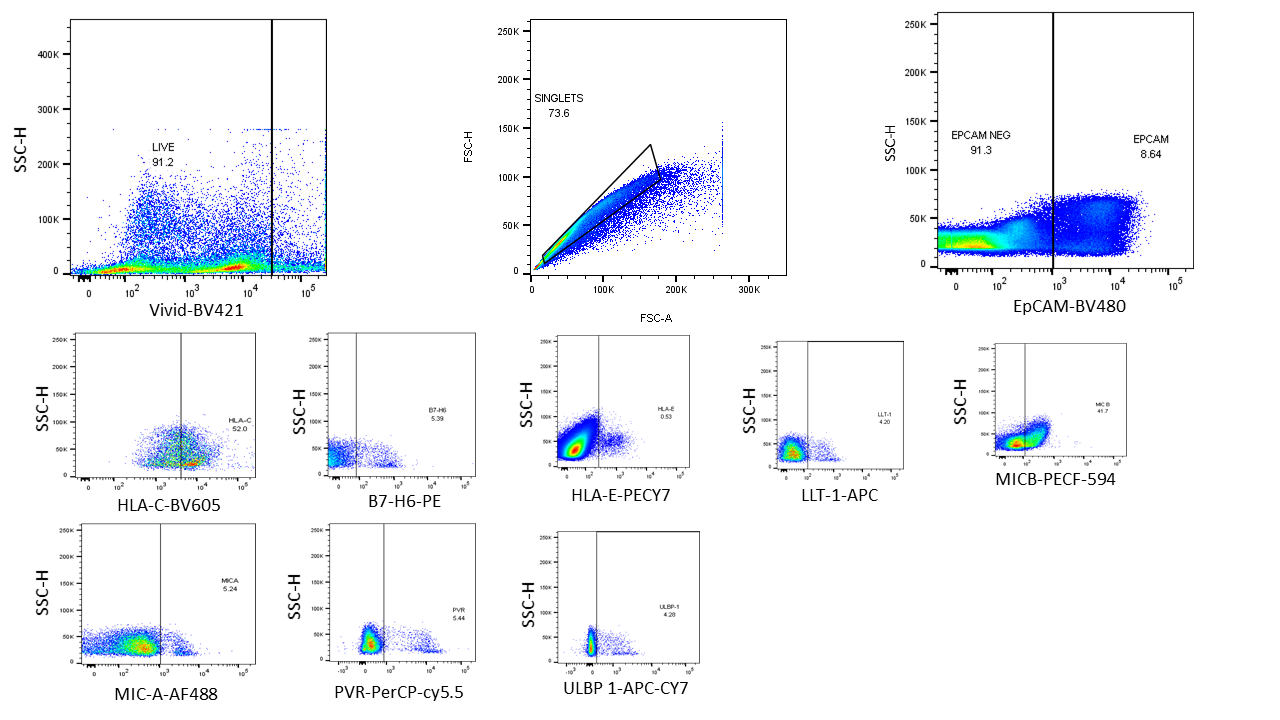


**Supplementary Figure 2** Representative gating strategy for ligands panel

Supplement: Supplementary file 2 — Additional file 2: Supplementary Figure S2. Representative gating strategy for ligands panel. [file 13048_2023_1192_MOESM2_ESM.docx]
