# Supplementary material for: Immune profile of primary and recurrent epithelial ovarian cancer cases indicates immune suppression, a major cause of progression and relapse of ovarian cancer
Source: J Ovarian Res. 2023 Jun 15;16:114. doi: 10.1186/s13048-023-01192-4 (PMC10268537; doi:10.1186/s13048-023-01192-4)
Supplement: Supplementary file 3 — Additional file 3: Supplementary Figure 3. Phenotype of tumor infiltrating (a-c) CD56Dim NK, NKT-like and T cell in pEOC and rEOC patients. [file 13048_2023_1192_MOESM3_ESM.docx]

A


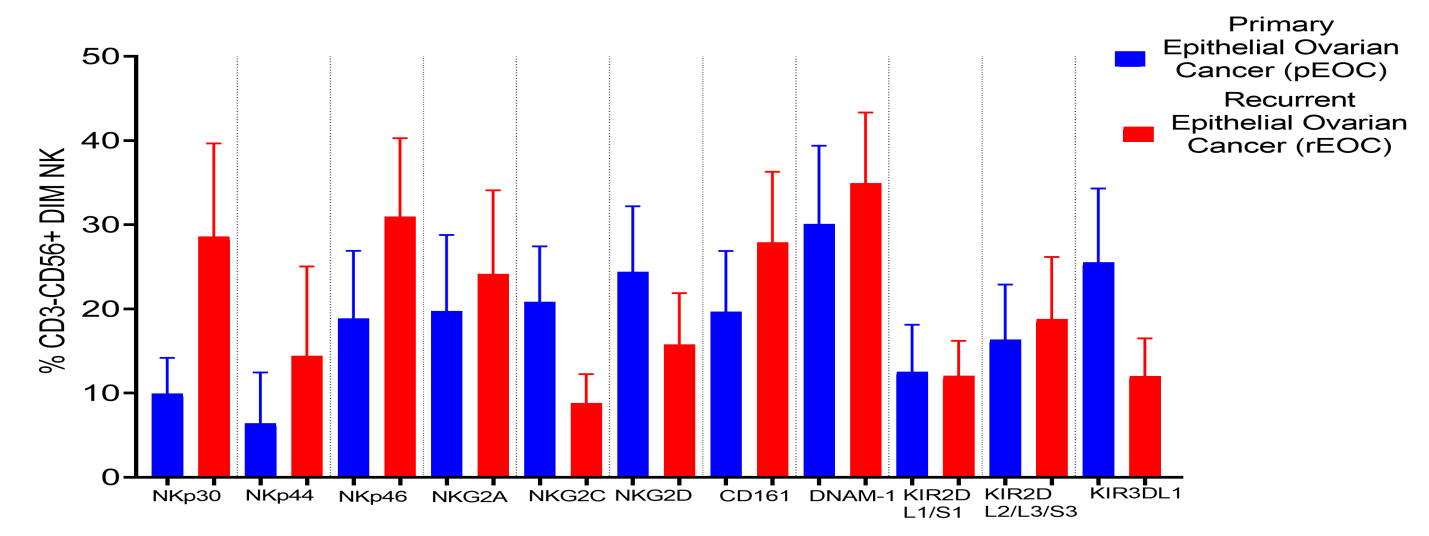


B


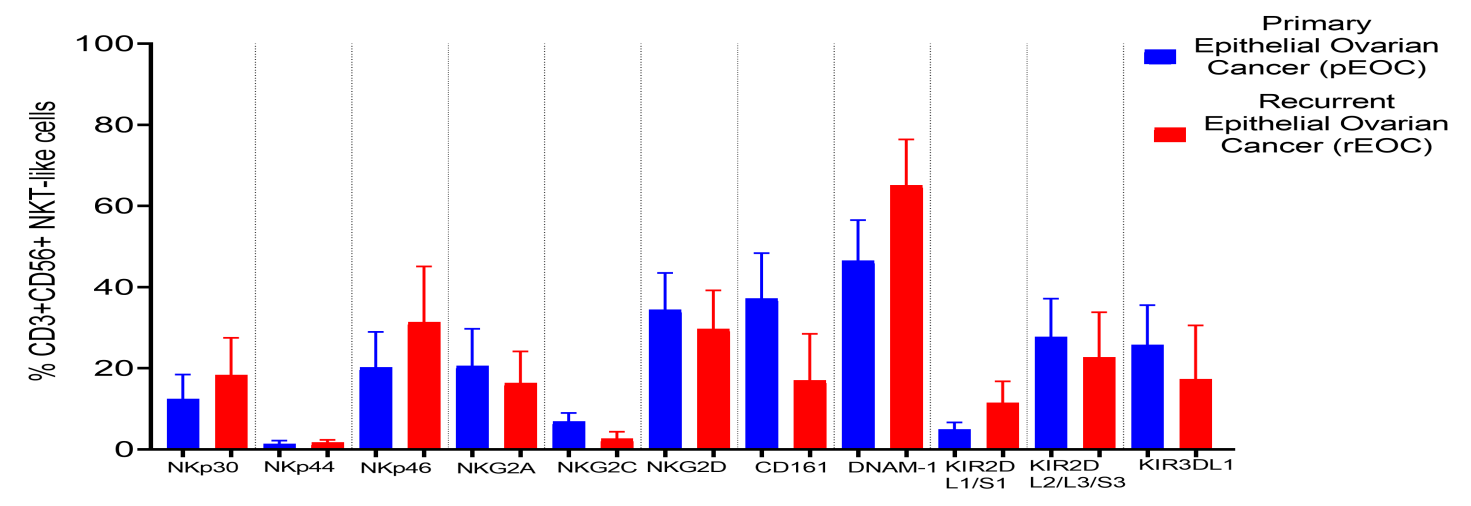


C


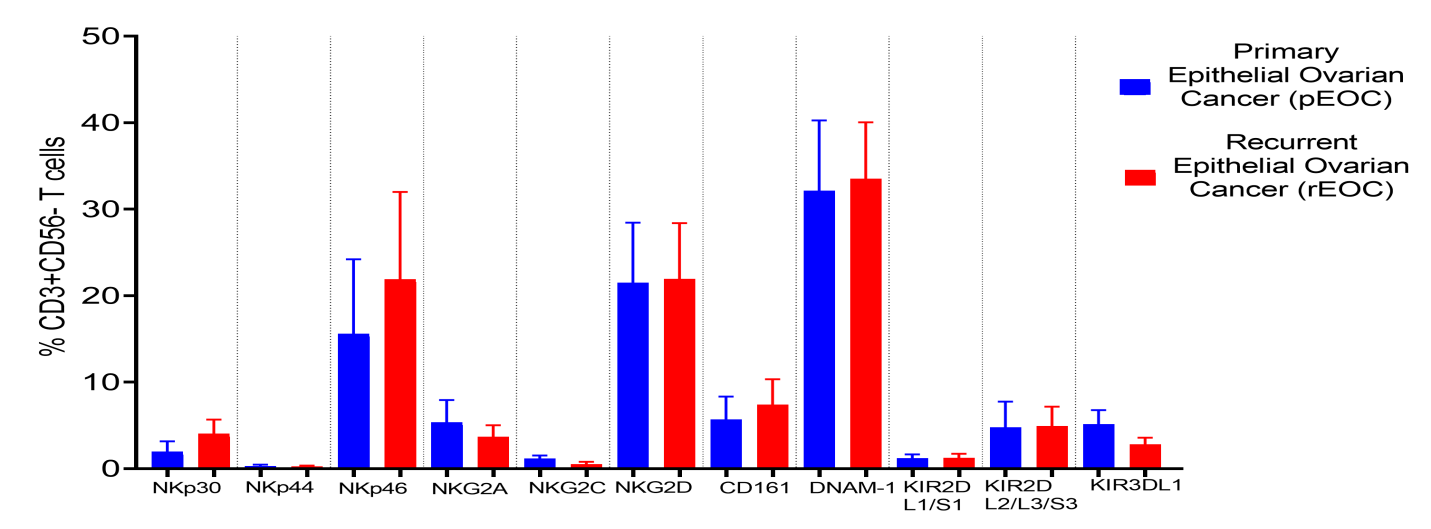


**Supplementary Figure 3** Phenotype of tumor infiltrating (a-c) CD56^Dim^ NK, NKT-like and T cell in pEOC and rEOC patients
