# Supplementary material for: Immune profile of primary and recurrent epithelial ovarian cancer cases indicates immune suppression, a major cause of progression and relapse of ovarian cancer
Source: J Ovarian Res. 2023 Jun 15;16:114. doi: 10.1186/s13048-023-01192-4 (PMC10268537; doi:10.1186/s13048-023-01192-4)
Supplement: Supplementary file 4 — Additional file 4: Supplementary Figure 4. Percentage of tumor cells positive for cognate ligands of NK cell receptors in tissue specimens of pEOC and rEOC patients. [file 13048_2023_1192_MOESM4_ESM.docx]

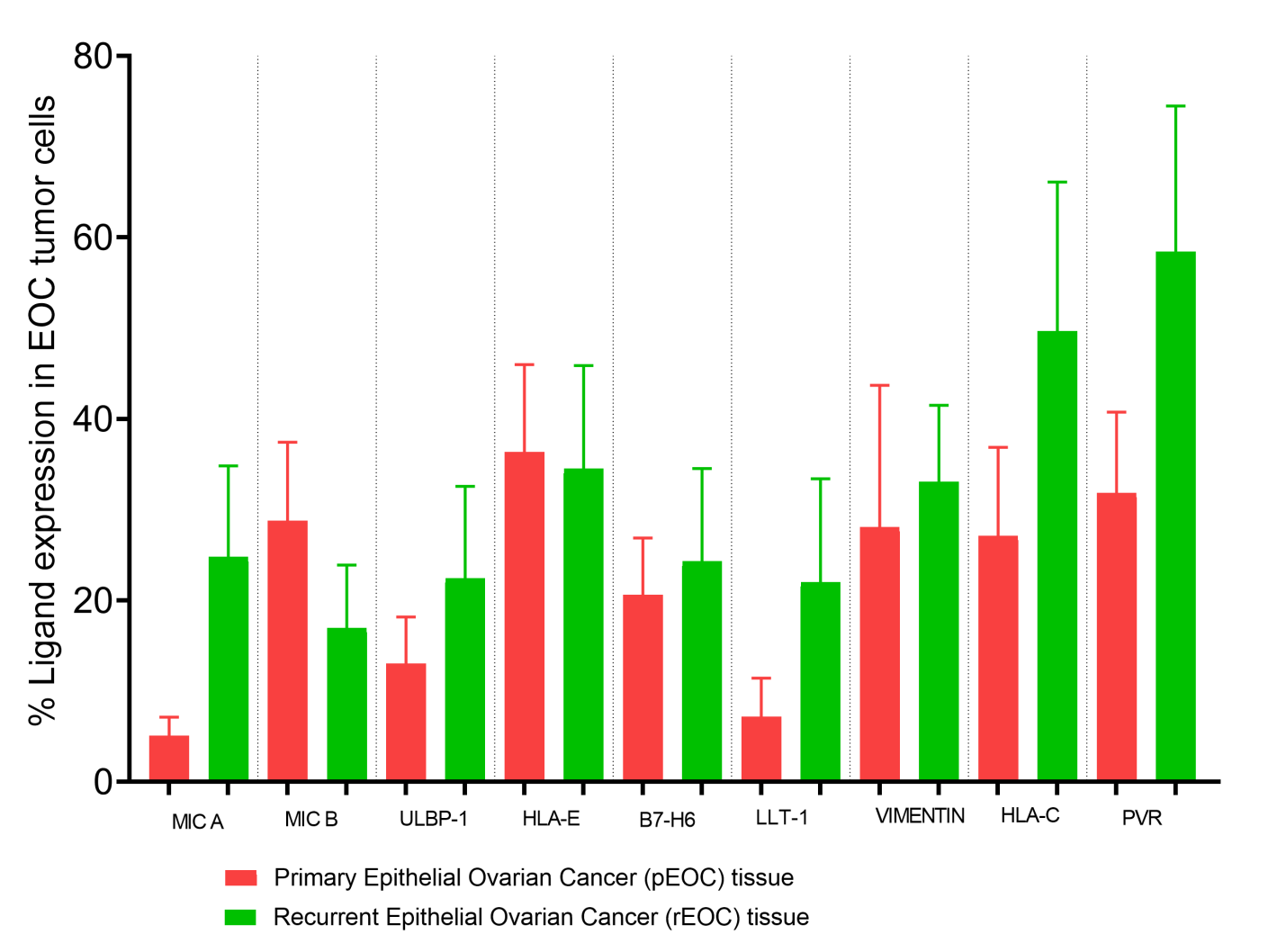


**Supplementary Figure 4** Percentage of tumor cells positive for cognate ligand of NK cell receptors in tissue specimens of pEOC and rEOC patients
