# Supplementary material for: Immune profile of primary and recurrent epithelial ovarian cancer cases indicates immune suppression, a major cause of progression and relapse of ovarian cancer
Source: J Ovarian Res. 2023 Jun 15;16:114. doi: 10.1186/s13048-023-01192-4 (PMC10268537; doi:10.1186/s13048-023-01192-4)
Supplement: Supplementary file 5 — Additional file 5: Supplementary Table S1. Details of antibodies used to stain NK cells receptors and ligands. [file 13048_2023_1192_MOESM5_ESM.docx]

| Panel for NK cell receptors | | | | Ligand Panel | |
| --- | --- | --- | --- | --- | --- |
| Panel 1  Fluorescent labeled antibody | Clone | Panel 2  Fluorescent labeled antibody | Clone | Fluorescent labeled antibody | Clone |
| Vivid dye- BV421 |  | Vivid-Dye-BV421 |  | Vivid-dye BV421 |  |
| CD45-PECY5.5 | HI30 | CD45-PECY5.5 | HI30 | EpCAM-BV480 | EBA-1 |
| CD56-PE | 5.1H11 | CD56-PE | 5.1H11 | B7-H6-PE | 875001 |
| CD3-PECF594 | UCHT1 | CD3-PECF594 | UCHT1 | MICA-AF488 | 159227 |
| NKG2D-APC-CY7 | 1D11 | NKp44-PECY7 | P44-8 | LLT-1 APC | 402659 |
| CD161-BV480 | DX12 | NKG2C-AF488 | 134522 | ULBP-1 AF750 | 170818 |
| DNAM-1-BV786 | 11A8 | KIR2DL1/S1-APC | EB6B | MICB-AF594 | 236511 |
| NKp30-BV605 | p30-15 | KIR3DL1-BV786 | DX9 | HLA-C-BV605 | DT-9 |
| NKp46-PerCP-cy5.5 | 9E2 | - | - | PVR-PerCP/CY5.5 | SKIL4 |
| NKG2A-APC | 131411 | - | - | HLA-C PECY7 | 3D12 |
| KIR2DL2/L3/S3-AF488 | 180704 | - | - | - | - |

**Supplementary Table S1** Details of antibodies used to stain NK cells receptors and ligands
