# Supplementary material for: Immune profile of primary and recurrent epithelial ovarian cancer cases indicates immune suppression, a major cause of progression and relapse of ovarian cancer
Source: J Ovarian Res. 2023 Jun 15;16:114. doi: 10.1186/s13048-023-01192-4 (PMC10268537; doi:10.1186/s13048-023-01192-4)
Supplement: Supplementary file 6 — Additional file 6: Supplementary Table S2. Spearman’s correlation between serum cytokine and receptor expression profile of CD56BrightNK, CD56DimNK, NKT-like, and T cells of healthy control, pEOC and rEOC patients. [file 13048_2023_1192_MOESM6_ESM.docx]

| Spearman’s correlation | | | | | | |
| --- | --- | --- | --- | --- | --- | --- |
| Cell Subset | Control group | | Primary CaOv | | Recurrent CaOv | |
| **CD56 B NK** | p | r | p | r | p | r |
| NKp30 Vs IL-8 | **0.0558** | -0.4901 | 0.1930 | 0.3840 | 0.5765 | 0.1909 |
| NKp44 Vs IL-2 | **0.0380** | 0.5240 | 0.5940 | -0.1540 | 0.7315 | 0.1095 |
| NKp44 Vs IL-8 | 0.4276 | -0.2119 | **0.0150** | 0.6440 | 0.1095 | -0.2563 |
| NKp46 Vs IFN-У | 0.1322 | 0.3581 | **0.0410** | 0.5550 | 0.7594 | 0.0862 |
| NKp46 Vs IL-10 | 0.1064 | -0.3822 | **0.0210** | 0.6160 | 0.5123 | 0.1825 |
| NKp46 Vs IL-6 | 0.6140 | 0.1237 | **0.0150** | 0.6400 | 0.7435 | -0.0929 |
| NKG2A Vs IL-8 | **0.0233** | -0.0233 | 0.8810 | -0.5000 | 0.6821 | 0.1515 |
| NKG2A Vs IL-6 | **0.0246** | -7.7220 | 0.2440 | 0.3620 | 0.4069 | 0.2970 |
| NKG2D Vs IL-10 | **0.0218** | -0.5224 | 0.4590 | 0.1910 | 0.7700 | 0.0823 |
| NKG2D Vs IL-2 | **0.0230** | 0.5183 | 0.5864 | -0.1410 | 0.8846 | -0.0414 |
| NKG2D Vs IL-8 | 0.3050 | 0.2480 | **0.0130** | 0.5950 | 0.6763 | -0.1179 |
| DNAM-1 Vs IL-8 | **0.0220** | -0.5050 | 0.9720 | 0.0110 | 0.7752 | -0.0804 |
| DNAM-1 Vs IL-6 | **0.0007** | -0.6920 | 0.6780 | -0.1200 | 0.3378 | -0.2645 |
| CD161 Vs IFN-У | **0.0479** | 0.4720 | 0.2760 | 0.2880 | 0.3565 | 0.2549 |
| CD161 Vs IL-10 | **0.0348** | -0.4990 | 0.3610 | -0.3614 | 0.1281 | -0.4114 |
| CD161 Vs IL-2 | **0.0320** | 0.5063 | 0.7130 | -0.9900 | 0.2863 | 0.2934 |
| **CD56 D NK** |  |  |  |  |  |  |
| NKG2A Vs IFN-У | **0.0580** | 0.4193 | 0.6410 | -0.1410 | 0.6095 | 0.18460 |
| NKG2C Vs TNF-α | **0.0220** | -0.4830 | 0.4920 | -0.2430 | 0.0794 | 0.72070 |
| NKG2D Vs IL-8 | 0.9662 | 0.0096 | **0.0170** | 0.5930 | 0.5640 | -0.16090 |
| DNAM-1 Vs IL-6 | **0.0100** | -0.5330 | 0.3510 | 0.2670 | 0.7925 | -0.07500 |
| DNAM-1 Vs TNF-α | **0.0300** | -0.4622 | 0.7960 | 0.0750 | 0.6470 | -0.12900 |
| DNAM-1 Vs IL-8 | 0.4743 | -0.1609 | **0.0160** | 0.6380 | 0.5320 | -0.17500 |
| KIR2DL2/L3/S3 Vs IL5 | **0.0067** | 0.5726 | 0.3210 | 0.2960 | 0.7621 | 0.09408 |
| KIR2DL2/L3/S3 Vs TNF-α | **0.0730** | -0.3890 | 0.6550 | 0.1350 | 0.4092 | -0.24900 |
| KIR3DL1 Vs IL-2 | **0.0230** | 0.4800 | 0.8810 | -0.0400 | 0.3276 | -0.27000 |
| **NKT-like cells** |  |  |  |  |  |  |
| NKG2D Vs IL-8 | 0.9659 | 0.01053 | **0.009** | 0.634 | 0.4657 | -0.2036 |
| CD161 Vs IL-8 | 0.7726 | -0.07327 | **0.011** | 0.625 | **0.0232** | -0.5893 |
| KIR3DL1 Vs IL-8 | **0.0007** | -0.712 | 0.998 | -0.001 | 0.2425 | 0.3214 |
| **T cells** |  |  |  |  |  |  |
| NKG2A Vs IFN-У | **0.0401** | -0.7470 | 0.6020 | -0.1580 | 0.8708 | 0.0616 |
| CD161 Vs IL-6 | **0.0122** | -0.5760 | 0.4390 | 0.2060 | 0.7043 | -0.1121 |
| CD161 Vs IL-8 | **0.0070** | -0.6120 | 0.1010 | 0.4250 | 0.1553 | -0.4022 |
| CD161 Vs IL-10 | 0.8110 | -0.0607 | 0.6856 | -0.1238 | **0.0187** | -0.6501 |
| KIR2DL2/L3/S3 Vs IL-2 | **0.0390** | -0.6720 | 0.6770 | 0.1260 | 0.7183 | 0.1094 |
| KIR2DL2/L3/S3 Vs IL-6 | **0.0290** | -0.7000 | 0.6780 | -0.1250 | 0.6856 | -0.1238 |

**Supplementary Table S2** Spearman’s correlation between serum cytokine and receptor expression profile of CD56^Bright^NK, CD56^Dim^NK, NKT-like, and T cells of healthy control, pEOC and rEOC patients
